# The complete chloroplast genome of *Philadelphus pekinensis* Rupr. (Hydrangeaceae)

**DOI:** 10.1080/23802359.2020.1730725

**Published:** 2020-02-27

**Authors:** Qin Fan, Mimi Li, Hui Zhou, Wanzhen Liu, Wei Gong

**Affiliations:** a College of Life Sciences, South China Agricultural University, Guangzhou, China;; b Institute of Botany, Jiangsu Province and Chinese Academy of Sciences, Nanjing, China

**Keywords:** *Philadelphus pekinensis*, chloroplast genome, ornamental plant, Illumina sequencing

## Abstract

*Philadelphus pekinensis* Rupr. is a common perennial deciduous shrub distributed in temperate China. Here, we report the complete chloroplast genome of *P. pekinensis*. The cp genome is totally 157,308 bp in length, including large single-copy (LSC) region of 86,457 bp, small single-copy (SSC) region of 18,735 bp, and two separated inverted regions (IRs) of 26,058 bp, respectively. It contains 134 genes, including 85 protein-coding genes, 8 rRNA genes, and 37 tRNA genes. The overall GC content is 43.1%. The phylogenetic analysis reveals the monophyly of *P. pekinensis* and *P. calvescens*, which is more related with *Carpenteria californica* than other species in the Hydrangeaceae family.

*Philadelphus pekinensis* Rupr. is famous for its beautiful, fragrantand long term flowers, making it as an ornamental shrub commonly cultivated in the botanical gardens. It grows in temperate China, Inner Mongolia, Korea, as well as Europe and North America. Here, we report the first chloroplast (cp) genome of *P. pekinensis* for further research on the phylogenetic and biogeographic study among its related species in the future.

Samples of *P. pekinensis* were collected from Yunmeng Mountain National Forest Park in Miyun District, Beijing (Voucher No.: LP161571). Total genomic DNA was extracted from silica gel dried leaves using a modified CTAB method (Doyle and Doyle 1987). The paired-end (2 × 150 bp) library was constructed by Illumina PE150 in Novogene Co. Ltd (Beijing, China). Totally, 2.64 Gb clean data were obtained after removing low-quality reads and adaptor sequences. The complete cp genome of *P. pekinensis* was assembled via GetOrganelle pipeline (available online: https://github.com/Kinggerm/GetOrganelle, JianJun et al. 2018), which implements four steps of recruiting plastid-like reads: (i) conducting *de novo* assembly using SPAdes (Anton et al. 2012), (ii) filtering plastid-like contigs, (iii) visualizing and editing *de novo* assembly graph using Bandage (Ryan et al. 2015), and (iv) checking and adjusting the annotations using Geneious 11.0.3 (http://www.geneious.com, Kearse et al. 2012). The tRNA genes were annotated with ARAGORN (Laslett and Canback 2004). For phylogenetic reconstruction, we downloaded the cp genomes of 10 species in the Hydrangeaceae family, including *Hydrangea densifolia* (MN380652), *Decumaria barbara* (MN380684), *D. sinensis* (MN380685), *Schizophragma hydrangeoides* (KY412467), *Deutzia crassifolia* (MG524993), *D. compacta* (MN380704), *Philadelphus calvescens* (MN380700), *Kirengeshoma palmata* (MN380656), *Carpenteria californica* (MN380687), and *Whipplea modesta* (MN380692). *Mukdenia rossii* (MG470844) from Saxifragaceae was used as an outgroup. The aligned matrix was implemented in MAFFT (Katoh and Standley 2013). Phylogenetic analysis was performed with maximum likelihood (ML) method, using RAxML (Stamatakis 2006; 2014) with 1000 bootstrap replicates.

The annotated cp genomic sequences of *P. pekinensis* were deposited in GenBank (MN938497). The whole cp genome is 156,876 bp in length and has the typical quadripartite structure, including large single-copy (LSC) region of 86,457 bp, small single-copy (SSC) region of 18,735 bp, and two separated inverted region (IRs) of 26,058 bp. A total of 134 genes are identified consisting of 85 protein-coding genes (PCGs), 8 rRNA genes, and 37 tRNA genes. Among these genes, 61 PCGs and 22 tRNA genes are located in the LSC region (including one interregional gene *rps19*), while 12 PCGs and 1 tRNA gene occur in the SSC region (including one interregional gene *ycf1*). All the eight rRNA genes are duplicated in the IR regions. Each of the IR regions contains six PCGs and seven tRNA genes. Each of the 19 genes contains one intron, while the remaining three (*ycf3*, *clpP,* and *rps12*) possess two introns each. The overall GC content is 43.1%. A well supported phylogenetic tree is reconstructed, suggesting a monophyly formed by *P. pekinensis* and *P. calvescens* (Figure 1). The two species demonstrate a comparably closer phylogenetic relationship with *Carpenteria californica* than other species.

**Figure 1.**
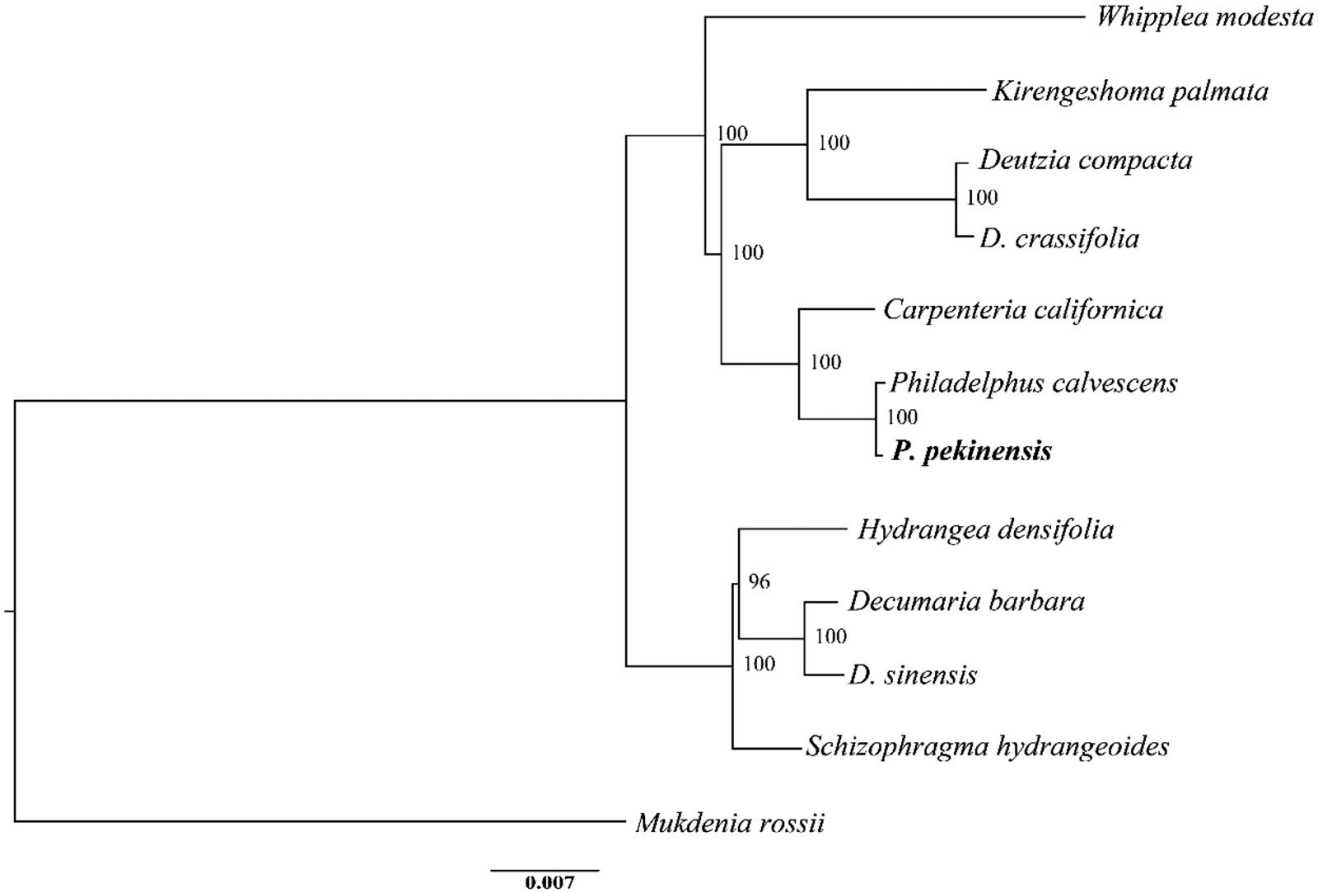
Maximum likelihood tree based on 11 complete chloroplast genomes of Hydrangeaceae and one outgroup species. Numbers in the nodes are bootstrap support values based on 1000 replicates. *Philadelphus pekinensis* is displayed in bold.
